# A Greek multicenter study comparing the clinical and immunologic phenotypes between adult and juvenile- onset lupus

**DOI:** 10.1186/1546-0096-9-S1-P245

**Published:** 2011-09-14

**Authors:** P Nalbanti, S Stefanidou, S Alfantaki, A Siamopoulou, M Trachana, V Galanopoulou, P Pratsidou-Gerts, E Farmaki, F Papachristou, A Garyphallos, F Kanakoudi-Tsakalidou

**Affiliations:** 1 1st Dept of Pediatrics, Aristotle University, Pediatric Immunology and Rheumatology Referral Center, Thessaloniki; 2 4th Dept of Internal Medicine, Aristotle University, Thessaloniki; 3 Dept of Child Health, University of Ioannina, Ioannina; 4 Rheumatology Unit, N. Papageorgiou Hospital, Thessaloniki, Greece

## Background and Aim

To compare the clinical and immunologic phenotypes between Juvenile and Adult onset SLE (jSLE, aSLE) in Greek patients at disease onset and 5 yrs thereafter due to paucity of relevant data from Mediterranean countries.

## Methods

This retrospective study enrolled 66 jSLE and 97 aSLE patients, all having been attended in 4 Rheumatology Centers. Demographic data, as well as clinical and immunologic findings at diagnosis and 5 yrs thereafter were studied.

## Results

At diagnosis the mean (SD) ages were 12.25(0.27) and 33.93(1.32) yrs, whereas the mean follow-up 6.63 (0.59) and 11.6 (0.7) yrs, for jSLE and aSLE respectively. General features, hepatosplenomegaly, lymphadenopathy and haematology abnormalities were more frequent in jSLE patients (p1=0.001, p2=0.025 and p3<0.0001, respectively), whereas photosensitivity was commoner in those with aSLE (p=0.047). The main difference was the higher mean number of organ/system involvement in the jSLE group (p=0.0006). At the end of 5 yrs, the cumulative number of clinical manifestations was similar in both groups. Anti-dsDNAs, anti-cardiolipin, anti-Sm, anti-URNP antibodies and low C3 and C4 were significantly commoner in jSLE (p<0.01).

## Conclusions

Findings of this study are in line with previous publications regarding the need for early aggressive therapy in jSLE due to the graver presentation at onset as compared to aSLE. However, the incidence of nephritis or CNS or skin involvement was not higher compared to aSLE, as previously reported. Moreover, no significant differences in clinical or immunologic phenotypes were cumulatively found between the 2 groups during the first 5 yrs of disease course.

